# Hydro-Ethanolic Extract of *Portulaca Oleracea* Ameliorates Total and Differential WBC, Lung Pathology and Oxidative Biomarkers in Asthmatic Rats

**DOI:** 10.22037/ijpr.2019.13712.11817

**Published:** 2019

**Authors:** Mohammad Hossein Boskabady, Mahsa Kaveh, Kaveh Shakeri, Nama Mohammadian Roshan, Ramin Rezaee

**Affiliations:** a *Neurogenic Inflammation Research Center, Mashhad University of Medical Sciences, Mashhad, Iran. *; b *Department of Physiology, School of Medicine, Mashhad University of Medical Sciences, Mashhad, Iran. *; c *Natural Products and Medicinal Plants Research Center, North Khorasan University of Medical Sciences, Bojnurd, Iran. *; d *Department of Pathology, School of Medicine, Mashhad University of Medical Sciences, Mashhad, Iran. *; e *Clinical Research Unit, Faculty of Medicine, Mashhad University of Medical Sciences, Mashhad, Iran.*

**Keywords:** Portulaca oleracea, WBC, Oxidant and antioxidant levels, Lung inflammation, Asthmatic rats

## Abstract

The effects of *Portulaca oleracea* (*P.* *oleracea*; PO) on total and differential WBC count, and oxidant/antioxidant biomarkers in bronchoalveolar lavage fluid (BALF) as well as on lung pathology in asthmatic rats were examined. Rats were randomly divided into; control group (C), asthma group, asthma groups treated with either *P. oleracea* (rats that received PO 1, 2 and 4 mg/mL) or dexamethasone 1.25 μg/mL (D), (n = 8 in each group). Total and differential white blood cells (WBC) count, nitrite (NO_2_), nitrate (NO_3_), malondialdehyde (MDA), superoxide dismutase (SOD), catalase (CAT) and thiol levels in rats BALF were evaluated and lung pathological features were studied. Total WBC count, eosinophil, neutrophil and monocyte percentages, levels of NO_2_, NO_3_, MDA in the BALF and most pathological scores in the lung were increased but lymphocyte percentage, SOD, CAT and thiol levels were decreased in the BALF of asthmatic animals (*p* < 0.05 to *p *< 0.001). Treatment with *P.* *oleracea* significantly reduced total WBC, neutrophil, eosinophil, monocyte, NO_2_, and NO_3_, MDA, interstitial fibrosis, emphysema, interstitial inﬂammation and epithelial damage, but increased lymphocyte, SOD, CAT and thiol levels compared to asthma group (*p *< 0.05 to *p *< 0.001). Dexamethasone-treated rats also showed significant improvements in most parameters compared to asthma group (*p* < 0.05 to *p *< 0.001). Our results demonstrated the ameliorative effects of *P.* *oleracea* on total and differential WBC count and oxidant-antioxidant biomarkers levels in BALF as well as lung pathological features in asthmatic rats, which propose the usage of this extract as a preventive anti-inflammatory treatment against asthma.

## Introduction

*Portulaca oleracea* (*P.* *oleracea*) commonly known as “purslane”, is an annual plant with yellow flowers and smooth, reddish, mostly prostrate stems and alternate leaves, which belongs to the Portulacaceae family and grows in the Middle East, India, Europe, Africa, North America, Australia, and Asia (1). *P. oleracea* contains potassium, magnesium, calcium, omega-3 fatty acid, alpha-linolenic acid, gamma-linolenic acid, alpha-tocopherol and ascorbic acid (2). Several therapeutic effects such as bronchodilatory (3), muscle relaxant (4), antioxidant (5), anti-tussive (6) and anti-inflammatory (7) activities were reported for this plant. Consistently, our previous studies demonstrated antioxidant, anti-inflammatory and immunomodulatory effects for *P. oleracea* in ovalbumin (OVA)-sensitized rats (8, 9). Aqueous extract of *P. oleracea* has been demonstrated to inhibit intracellular reactive oxygen species (ROS) production and NF-κB activation in vascular endothelial cells (10). 

Asthma is a chronic inflammatory disease that is defined as reversible airway obstruction, bronchial hyperresponsiveness and increased airway inflammation, inflammatory cells infiltration, and mucus production (11, 12). Inflammatory cells such as T cells, mast cells, basophils, macrophages, and eosinophils are involved in the inflammatory processes underlying asthma (13). Concerning these cells, increased number of eosinophils is a characteristic feature of asthma (14). In this regard, several studies indicated that total WBC and eosinophil counts were enhanced in asthmatic animals and patients (15, 16). Exposure to allergens and respiratory infections causes migration of inflammatory cells and production of inflammatory mediators (17). Increased levels of inﬂammatory mediators such as ROS, histamine and products of arachidonic acid metabolism, lead to airway remodeling and pathological features such as mucus hypersecretion, smooth muscle hyperplasia, sub-epithelial fibrosis, thickening of the airway walls, inﬁltration of inﬂammatory cells, increased smooth muscle mass, vascular congestion and airway epithelial shedding (18). Structural changes in the airways induce irreversible airflow obstruction and airway hyperresponsiveness in asthma (19). 

Therefore, the present study aimed to evaluate the effect of hydro-ethanolic extract of *P. oleracea* on total and differential WBC, the levels of nitric oxide (NO), malondialdehyde (MDA), catalase (CAT), superoxide dismutase (SOD) and thiol in asthmatic rats BALF and study pathological features of their lungs.

## Experimental

*Plant collection and extraction*

*P. oleracea* was collected from Sabzevar city, Khorasan Razavi province, Iran, in July 2017. A voucher sample was kept at the herbarium of the School of Pharmacy, Mashhad University of Medical Sciences (Herbarium No. 240-1615-12). The leaves of *P. oleracea* were grounded to powder (100 g), mixed with 70% ethanol at a ratio of 1:10 (powder: ethanol) and left for 3 days at 37 °C with occasional shaking and stirring. The mixture was then filtered and the resulting liquid was concentrated under reduced pressure at 45 °C in an Eyela rotary evaporator (Heidolph, Germany). The yield of extraction was 17.5% (9). 

*Animals*

Experiments were performed using Wistar rats (200 ± 20 g) purchased from the animal house of School of Medicine, Mashhad University of Medical Sciences, Mashhad, Iran. The animals were kept in cages receiving clean filtered air (Maximiser, Thoren Caging System Inc., Hazleton, PA, U.S.A.) under standard conditions at 22 ± 2 ^o^C with regular 12 hr/ 12 hr light/dark cycles. They also had free access to food and water *ad libitum* during the experimental period.

*Animals sensitization*

A rat model of asthma was induced by three i.p. injections of 1 mg/kg of OVA, along with 0.9% sterile saline containing 100 mg Al(OH)_3_ as an adjuvant on days 1, 2 and 3 of the experiment. On days 6, 9, 12, 15, 18 and 21 of the experiment, animals were challenged with 1% OVA aerosol produced by a DeVilbiss PulmoSonic nebulizer (DeVilbiss Health Care Ltd., Feltham, U.K.) in a whole-body inhalation exposure chamber of 0.8 m^3^ for 20 min/day (16). 

*Experimental groups*

Animals were randomly divided into six groups (n = 8 in each group) including: 1) Control group (group C) which was given intra-peritoneal (i.p.) and inhaled normal saline; 2) Asthmatic group (group A) which was sensitized with OVA; 3) Asthmatic group treated with dexamethasone 1.25 μg/mL (group A+D); and 4-6) Asthmatic groups treated with *P. oleracea *extract 1, 2, and 4 mg/mL (groups A+PO 1, 2, and 4 mg/mL, respectively). *P. oleracea* extract and dexamethasone were added to animals’ drinking water during the 21-day sensitization period. Each animal consumed almost 40 ml water per day and the volume of consumption was not significantly different among different groups; thus, dexamethasone-treated rats received 50 μg/day and PO-treated rats received 40, 80 or 160 mg/day of the extract for 21 days.

*Collection of bronchoalveolar lavage fluid (BALF)*

All rats were sacrificed on day 22 by ketamine (50 mg/kg, i.p.), their chest was opened, and the trachea and lungs were removed. The left lung was lavaged five times with 1 mL saline (a total of 5 mL). Then, BALF was centrifuged at 2500 g at 4 °C for 10 min and supernatants were collected and stored at -80 °C until analysis. Total and differential WBC counts as well as oxidant/anti-oxidant biomarkers levels were then assessed in the BALF.

*Measurement of total and differential WBC count*

To count total leukocytes, 1 mL of BALF was stained with Turk’s solution (containing 1 mL glacial acetic acid, 1 mL gentian violet solution 1% and 100 ml pure water) and total WBC was determined in duplicate using a hemocytometer (in a Burker chamber). The rest of BALF was centrifuged at 2500 g at 4 °C for 10 min. For differential WBC count, a smear was prepared from the cell pellet in BALF and stained with Wright-Giemsa. After staining, differential counts were determined in accordance with standard morphologic protocols under a light microscope by counting a total of 100 cells/slide. Then, the percentage of each leukocyte was calculated (20).

*Measurement of oxidant and antioxidant levels*

Total stable oxidation products of NO me­tabolism (NO_2_^-^/NO_3_^-^) in BALF supernatant was evaluated using the Griess reagent containing sulfanilamide (SULF) and N-(1-Naph­thyl) ethylenediamine dihydrochloride (NEDD). The frozen BALF was thawed at 25˚ C, and deproteinized using zinc sulfate (Sigma, America). The liquefied BALF was then centrifuged at 12000 g for 10 min. Next, 300 μL of the clear supernatant was mixed with Griess reagent in water, in a test tube. For reduction of nitrate to ni­trite, 300 μL saturated solutions of vanadium (III) chloride (VCl_3_; Sigma, USA) in 1 M HCl was added to the mixture and incubated for 2 hr at 30 °C, in the dark. Then, the absorbance of samples was assessed at 540 nm against a blank containing the same concentrations of ingredients but no biological sample. Linear regression was used to determine NO concentration using a standard curve plotted for NaNO_2_. The final results were expressed as μmol (20).

Moreover, MDA levels, as an index of lipid peroxidation, was measured. MDA reacts with thiobarbituric acid (TBA) as a thiobarbituric acid reactive substance (TBARS) to produce a red complex with the maximum absorbance at 535 nm. For MDA measurement, 2 mL of TBA/trichloroacetic acid (TCA)/HCl was added to 1 mL of BALF supernatant and the mixture was heated in a water bath for 40 min. Then, the mixture was centrifuged at 1000 g for 10 min. Finally, the absorbance was measured at 535 nm (20). 

A colorimetric assay involving production of superoxide by pyrogallol autoxidation and prevention of diminution of the tetrazolium dye, MTT (3-(4,5-dimethylthiazol-2-yl)2,5-diphenyltetrazolium bromide) to formazan by SOD, was used and measured at 570 nm (20). One unit of SOD activity was defined as the quantity of enzyme required for prevention of MTT reduction rate by 50%.

Catalase (CAT) activity was assessed based on the rate constant *k* (dimension: S-1, k) of hydrogen peroxide decomposition. The reduction in absorbance at 240 nm per minute and the rate constant of the enzyme were determined. Activities were defined as k (rate constant) per liter (20). 

Total thiol concentration was measured using DTNB reagent which reacts with thiol moieties to produce a yellow complex with the maximum absorbance at 412 nm. Briefly, 1 mL tris-ethylenediaminetetraacetic acid (Tris-EDTA) buffer (pH 8.6) was added to 50 μL serum supernatant in 1-mL cuvettes and sample absorbance was read at 412 nm against Tris-EDTA buffer alone (A_1_). Then, 20 μL DTNB reagents (10 mmol/ L in methanol) was added to the mixture and after 15 min (at room temperature), the sample absorbance was read again (A_2_). The absorbance of DTNB reagent alone was also read as blank (B). Total thiol concentration (mmol/L) was calculated using the following equation (20):

Total thiol concentration (mmol/L) = (A_2_–A_1_–B)×1.07/0.05×13.6.

*Pathological evaluations*

After sacrificing the animals, lungs were removed and placed into buffered formalin 10% (Merck, Darmstadt, Germany). Seven days later, tissues were dried using Auto Technicon apparatus and cleared by passage of tissues through 70–100% ethanol and xylol and parafﬁn blocks were prepared. The specimens were cut into 4-μm slices and stained with hematoxylin and eosin (H&E). The tissues were then evaluated under a light microscope. In this study, we focused on the following pathological changes in the lungs of asthmatic and treated groups: interstitial inflammation, interstitial fibrosis, bleeding, emphysema and epithelial damage. Scoring of pathological changes was performed as previously explained: 0) No pathologic changes; 1) Patchy changes; and 2) severe changes (in most parts of the lung) (21).

*Statistical Analysis*

The data of total and differential WBC count, oxidant/antioxidant biomarkers levels and lung pathological studies were expressed as mean ± SEM. The comparisons of the data obtained from *P. oleracea*-treated groups, asthma group and control group were made using one way analysis of variance (ANOVA) with Tukey-Kramer’s post-test. Significance was accepted at *p *< 0.05.

**Figure.1:**
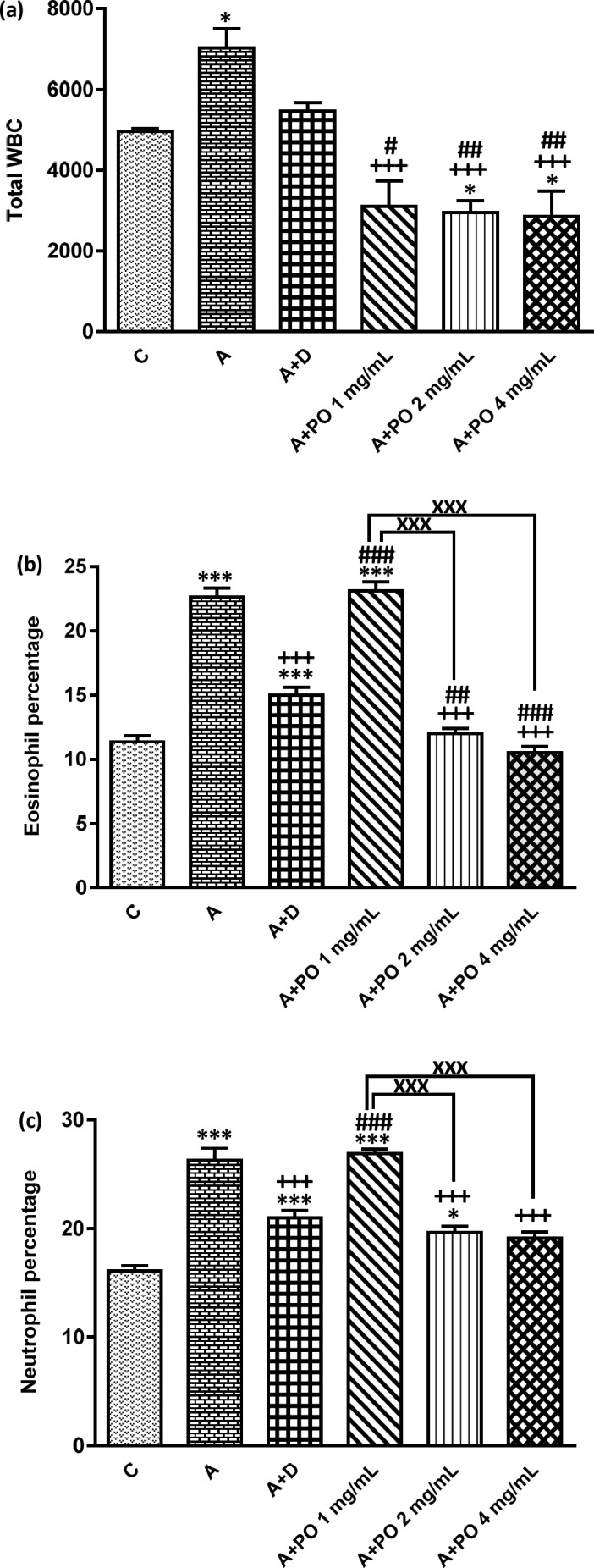
Total WBC number (count/ml bronchoalveolar lavage fluid (BALF)), (a), percentages of eosinophils (b) and neutrophils (c) in control animals (C), asthma group (A), asthmatic rats treated with dexamethasone (A+D) and asthmatic rats treated with *P. oleracea *(PO 1, 2 and 4 mg/mL) (n=8 in each group). Data are presented as mean ± SEM values. * *p* < 0.05 and *** *p *< 0.001 show significant differences compared to group C. +++ *p* < 0.001 shows significant differences compared to group A. # *p* < 0.05, ## *p *< 0.01 and ### *p *< 0.001 show significant differences compared to group A+D. xxx p < 0.001 shows significant differences among the three concentrations of *P. oleracea*. Statistical analyses were performed using one way analysis of variance (ANOVA) with Tukey-Kramer’s post-test

**Figure.2:**
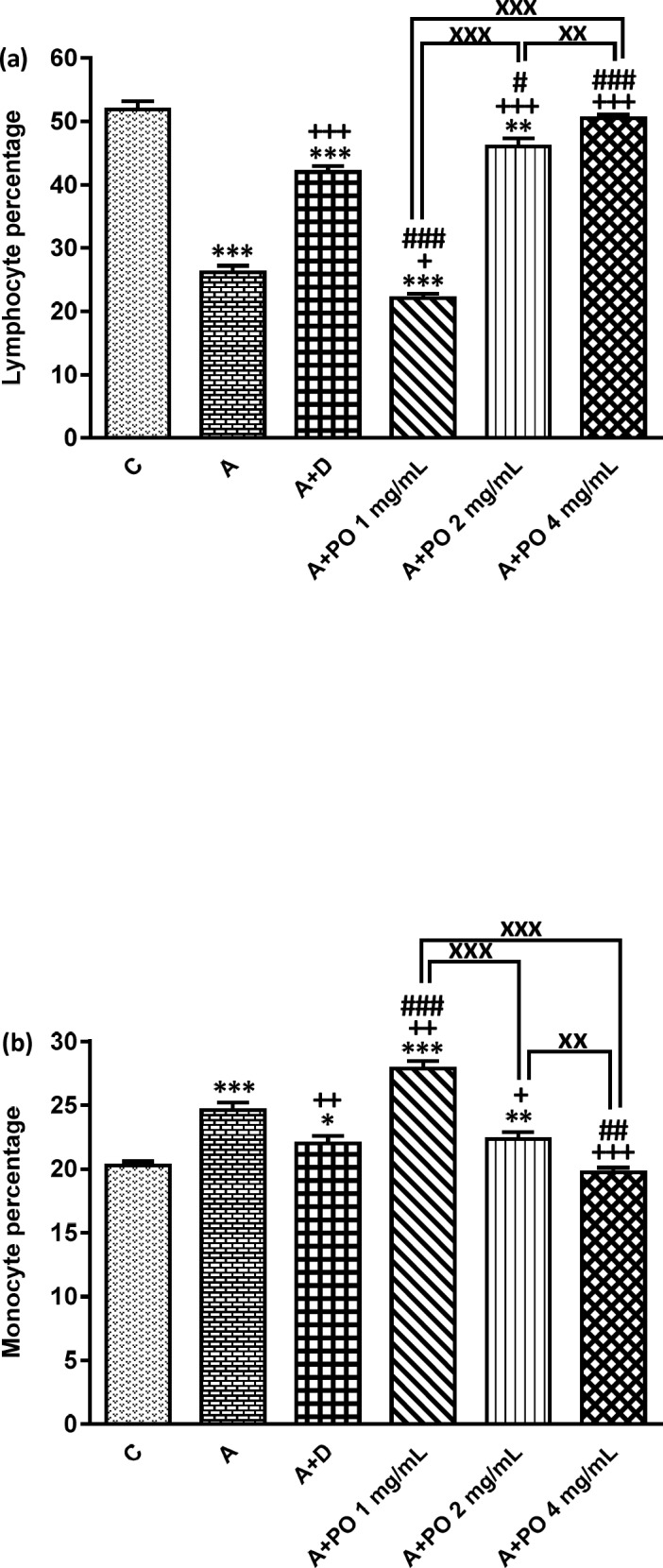
Percentages of lymphocyte (a) and monocyte (b) in bronchoalveolar lavage fluid (BALF) of control animals (C), asthma group (A), asthmatic rats treated with dexamethasone (A+D) and asthmatic rats treated with *P. oleracea *(PO 1, 2 and 4 mg/mL) (n = 8 in each group). Data are presented as expressed as mean ± SEM values. * *p *< 0.05, ** *p *< 0.01 and *** *p *< 0.001 show significant differences compared to group C. + *p *< 0.05, ++ *p *< 0.01 and +++ *p *< 0.001 show significant differences compared to group A. ## *p *< 0.05, ## *p *< 0.01 and ### *p *< 0.001 show significant differences compared to group A+D. xx *p *< 0.01 and xxx *p *< 0.001 show significant differences among the three concentrations of *P. oleracea*. Statistical analyses were performed using one way analysis of variance (ANOVA) with Tukey-Kramer’s post-test

**Figure 3:**
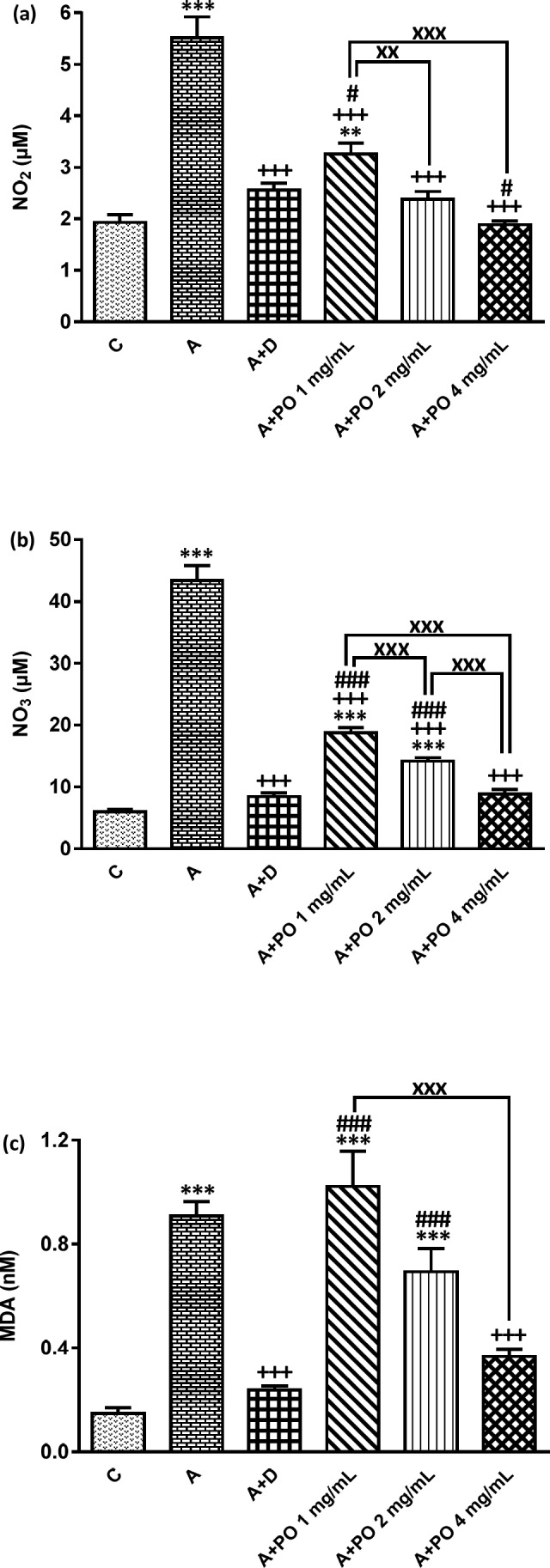
NO_2_ (a), NO_3_ (b) and MDA (c) concentration in bronchoalveolar lavage fluid (BALF) of control (C) asthma group (A), asthmatic rats treated with dexamethasone (A+D) and asthmatic rats treated with *P. oleracea *(PO 1, 2 and 4 mg/mL) (n = 8 in each group). Data are expressed as mean ± SEM values. ** *p* < 0.01 and *** *p *< 0.001 show significant differences compared to group C. +++ *p *< 0.001 shows significant differences compared to group A. # *p *< 0.05 and ### *p *< 0.001 show significant differences compared to group A+D. xx *p *< 0.01 and xxx *p *< 0.001 show significant differences among the three concentrations of *P. oleracea*. Statistical analyses were performed using ANOVA with Tukey-Kramer’s post-test

**Figure 4:**
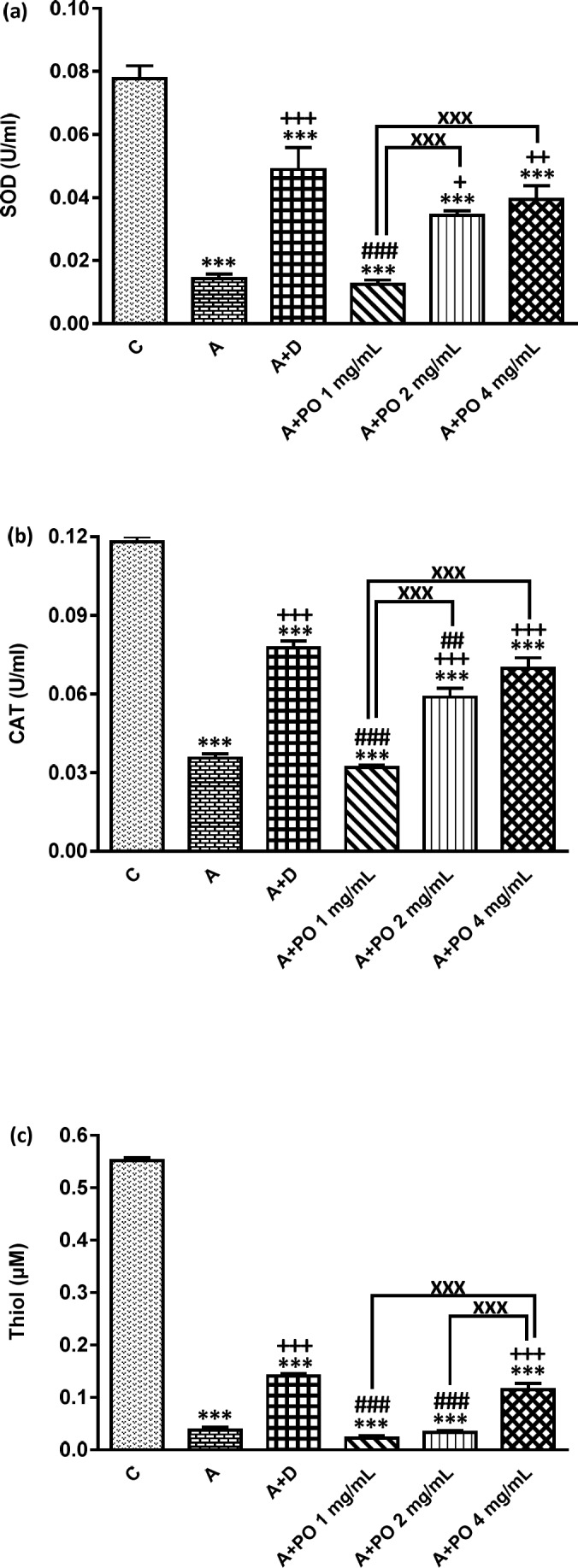
SOD (a), CAT (b) and Thiol (c) levels in bronchoalveolar lavage fluid (BALF) of control (C), asthma (A), asthmatic rats treated with dexamethasone (A+D) and asthmatic rats treated with *P. oleracea *(PO 1, 2 and 4 mg/mL) (n = 8 in each group). Data are expressed as mean ± SEM values. *** *p *< 0.001 shows significant differences compared to group C. + *p *< 0.05, ++ *p *< 0.01 and +++ *p *< 0.001 show significant differences compared to group A. ## *p *< 0.01 and ### *p *< 0.001 show significant differences compared to group A+D. xxx *p*<0.001 shows significant differences among the three concentrations *P. oleracea*. Statistical analyses were performed using ANOVA with Tukey-Kramer’s post-test

**Figure 5:**
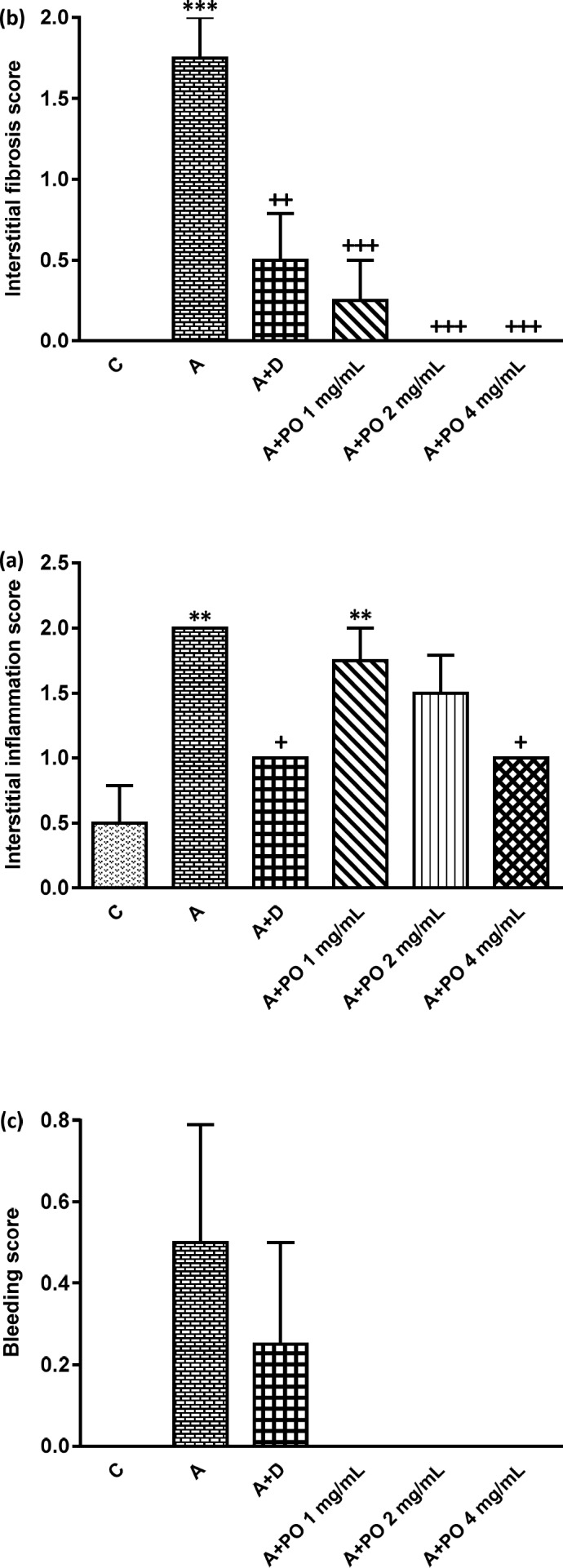
Interstitial inﬂammation (a), interstitial fibrosis (b) and bleeding (c) scores in control (C), asthma (A), asthmatic rats treated with dexamethasone (A+D) and asthmatic rats treated with *P. oleracea *(PO 1, 2 and 4 mg/mL) (n = 8 in each group). Data are expressed as mean ± SEM values. ** *p *< 0.01 and *** *p *< 0.001 show significant differences compared to group C. + *p *< 0.05, ++ *p *< 0.01 and +++ *p *< 0.001 show significant differences compared to group A. Statistical analyses were performed using ANOVA with Tukey-Kramer’s post-test

**Figure 6:**
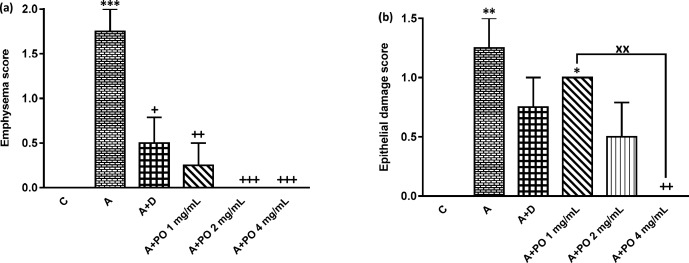
Emphysema (a) and epithelial damage (b) scores in control (C), asthma (A), asthmatic rats treated with dexamethasone (A+D) and asthmatic rats treated with *P. oleracea *(PO 1, 2 and 4 mg/mL) (n = 8 in each group). Data are expressed as mean ± SEM values. * *p *< 0.05, ** *p *< 0.01 and *** *p *< 0.001 show significant differences compared to group C. ++ *p *< 0.05, ++ *p *< 0.01 and +++ *p *< 0.001 show significant differences compared to group A. xx *p *< 0.01 shows significant differences among the three concentrations *P. oleracea*. Statistical analyses were performed using ANOVA with Tukey-Kramer’s post-test

**Figure. 7:**
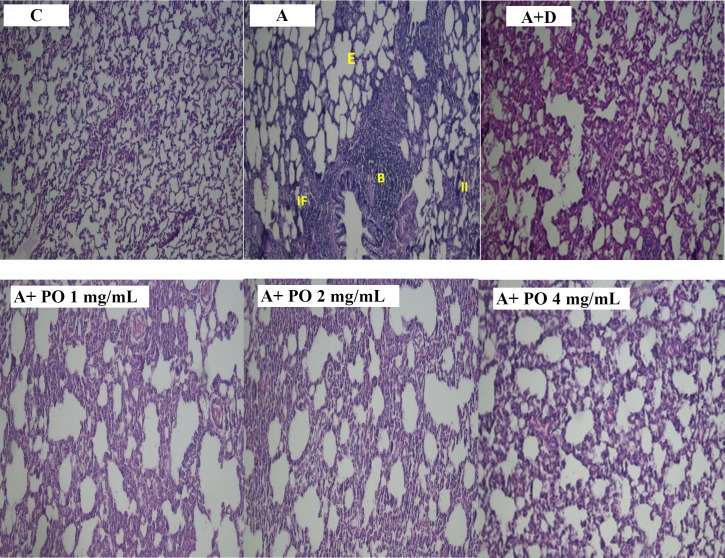
Pathological studies of lung specimens under a light microscope (X40), in control (C), asthmatic (A) group with interstitial inﬂammation (II), interstitial fibrosis (IF), bleeding (B) and emphysema (E), asthmatic rats treated with dexamethasone (A+D) and asthmatic rats treated with *P. oleracea *(PO 1, 2 and 4 mg/mL)

## Results

*Total and differential WBC count*

In asthmatic animals, total WBC count and the percentages of monocytes, eosinophils and neutrophils in the BALF were signiﬁcantly higher than those of the control group but lymphocyte percentage was lower compared to the control group (*p *< 0.05 for total WBC and *p *< 0.001 for other cases; Figures 1 and 2).

Total WBC counts in asthmatic animals treated with all concentrations of *P. oleracea* as well as the percentages of monocytes, eosinophils, neutrophils and lymphocytes in groups treated with the two higher concentrations of *P. oleracea* were significantly improved compared to those of untreated asthma group (*p *< 0.05 to *p *< 0.001; Figures 1 and 2).

Dexamethasone treatment also significantly reduced the percentages of monocytes, eosinophils and neutrophils but increased lymphocytes percentage compared to asthma group (*p *< 0.01 to *p *< 0.001; Figures 1 and 2).

However, the percentage of eosinophils in animals treated with 1 mg/mL of* P. oleracea*, the percentages of monocytes, neutrophils and lymphocytes in animals treated with the two lower concentrations of *P. oleracea* as well as total WBC count in groups treated with the two higher concentrations of *P. oleracea*, were significantly different from those of the control group (*p* < 0.05 to *p* < 0.001; Figures 1 and 2). 

All differential WBC counts in asthmatic animals treated with dexamethasone were also significantly different from those of the control group (*p *< 0.05 to *p *< 0.001; Figures 1 and 2). 

*Oxidant and antioxidant biomarkers*

The BALF levels of NO_2_, NO_3_ and MDA were significantly increased while SOD, CAT and thiol levels decreased in asthmatic animals compared to the control group (*p *< 0.001 for all cases; Figures 3 and 4).

NO_2_ and NO_3_ levels in asthmatic animals treated with all concentrations of *P. oleracea*, SOD and CAT levels in groups treated with the two higher concentrations and MDA and thiol levels in the group treated with the highest concentration of *P. oleracea*, were significantly improved compared to those of untreated asthma group (*p *< 0.05 to *p *< 0.001; Figures 3 and 4).

Dexamethasone treatment also significantly reduced NO_2_, NO_3_ and MDA but increased SOD, CAT and thiol levels compared to asthma group (*p *< 0.001 for all cases; Figures 3 and 

4).

SOD, CAT and thiol levels in groups treated with all concentrations of *P. oleracea*, NO_3_ and MDA levels in groups treated with the two lower concentrations and NO_2_ level in the group treated with the lowest concentration, were significantly different from those of the control group (*p *< 0.01 to *p *< 0.001; Figures 3 and 4).

The levels of all antioxidant biomarkers in dexamethasone-treated asthmatic animals were also significantly different from those of the control group (*p*<0.001 for all cases; Figures 3 and 4).

*Lung pathological studies*

The scores of all pathological changes except for bleeding, in asthma group were signiﬁcantly increased compared to the control group (*p *< 0.01 to *p *< 0.001; Figures 5 and 6).

Interstitial fibrosis and emphysema in asthma groups treated with all concentrations of *P. oleracea*, and interstitial inflammation and epithelial damage in the group treated with the highest concentration, were significantly reduced compared to untreated asthma group (*p* < 0.05 to *p *< 0.001; Figures 5 and 6).

Dexamethasone treatment also significantly reduced all pathological insults except for bleeding and epithelial damage compared to asthma group (*p *< 0.05 to *p *< 0.01; Figures 5 and 6).

Interstitial inflammation and epithelial damage scores in group treated with the lowest concentration of *P. oleracea* were significantly different from those of the control group (*p*<0.05 to *p*<0.01; Figures 5 and 6).

Overall, there was no significant difference in all pathological changes between the control and dexamethasone-treated group. A specimen photograph of lung histology in each studied group was presented in Figure 7. 

*Comparison of the effects of the extract and dexamethasone*

The effects of the lowest concentration of *P. oleracea* on all differential WBC counts were significantly less marked than those of dexamethasone (*p *< 0.001 for all cases; Figures 1 and 2). 

However, the effect of treatment with all concentrations of *P. oleracea* on total WBC, that of the two higher concentrations of *P. oleracea* on eosinophils and lymphocytes percentages, as well as the influence of the highest concentration of *P. oleracea* on monocytes percentage, were significantly higher than those of dexamethasone (*p*<0.05 to *p*<0.001; Figures 1 and 2).

The effect of the lowest concentration of *P. oleracea* on NO_2_ and SOD levels and its two lower concentrations on NO_3_, MDA, thiol and CAT levels were significantly less pronounced than those of dexamethasone (*p *< 0.05 to *p *< 0.001; Figures 3 and 4). However, the effects of the highest concentration of *P. oleracea* on NO_2_ level were significantly more marked than that of dexamethasone (*p *< 0.05; Figure 3).

There was no significant difference in all pathological scores among dexamethasone-treated group and groups treated with three concentrations of *P. oleracea.*

*Comparison of the effects of different concentrations of the extract*

The effects of two higher concentrations of *P. oleracea* (2 and 4 mg/mL) on monocytes, eosinophils, neutrophils and lymphocytes percentages were significantly more marked than the lowest concentration (1 mg/mL), (*p *< 0.001 for all cases; Figures 1 and 2). In addition, there was a significant difference between the effects of high and medium concentrations of *P. oleracea* on monocytes and lymphocytes percentages (*p *< 0.01 for both cases; Figures 1 and 2). 

The effects of two higher concentrations of *P. oleracea* (2 and 4 mg/mL) on all oxidant and antioxidant biomarkers levels except for the effect of the highest concentration (4 mg/mL) on MDA and thiol levels, were significantly more marked compared to the lowest concentration (1 mg/mL), (*p *< 0.01 to *p *< 0.001; Figures 3 and 4). The effect of *P. oleracea* 4 mg/mL on NO_3_ and thiol levels was also significantly more marked compared to *P. oleracea* 2 mg/mL (*p *< 0.001 for both cases; Figures 3 and 4).

Furthermore, *P. oleracea* 4 mg/mL had significantly more marked effects on epithelial damage compared to *P. oleracea* 1 mg/mL (*p *< 0.01; Figure 4).

## Discussion

*P. oleracea *has been used as an edible plant, and a traditional medicine for alleviating a wide spectrum of diseases such as gastrointestinal problems, respiratory diseases, hepatitis, interstitial cystitis, sleep disorders, fevers and headaches (22, 23). Also, anti-inflammatory and anti-bacterial properties of *P. oleracea *have been reported (7, 24). This plant has also shown anti-oxidant properties in a model of vitamin A deficiency which was suggested to be due to the presence of active constituents such as oleracein A, oleracein B and oleracein E (25). In an *in-vitro* study, *P. oleracea *increased cell survival and reduced IL-6 and TNF-α levels, the latter being known as a key pro-inflammatory mediator (26). Furthermore, in a recent study, oleracone, a novel alkaloid isolated from *P. oleracea* significantly reduced NO production, *in-vitro* (27).

In the present study, effects of *P. oleracea *extract on total and differential WBC count and levels of oxidant (NO_2_, NO_3_ and MDA) and antioxidant (CAT, SOD and thiol) biomarkers in BALF as well as lung pathological features were investigated in asthmatic rats. 

Based on our results, BALF of asthmatic animals had increased total WBC, eosinophils, neutrophils, monocytes counts but the lymphocytes percentage was reduced compared to non-asthmatic (control) animals. These results confirmed the induction of an experimental model of asthma which was also reported by previous studies (16, 20). Increased numbers of eosinophils, a prominent feature of asthma, have been observed in asthmatic patients and correlate with the severity of asthma which was also seen in the present study (28). It has been found that activated eosinophils play an important role in the inflammatory events involved in allergic asthma and airway hyperresponsiveness (29). Findings of this study showed that treatment of asthmatic animals with the extract of *P. oleracea *resulted in a significant reduction in total WBC, neutrophils, eosinophils and monocytes counts while it increased lymphocyte percentages. Consistently, a previous study on the effect of the hydro-ethanolic extract of *P. oleracea *on total and differential WBC count in sensitized rats reported comparable results (9).

In the current experiment, NO_3_, NO_2_ and MDA levels as indicators of oxidative stress were significantly increased but SOD, CAT and thiol levels decreased in asthmatic animals compared to the control group. Similarly, previous evidence also indicated an imbalance in oxidant/antioxidant enzymes balance towards oxidative conditions in sensitized animals (9, 20). Oxidative stress plays an important role in asthmatic airway inflammation, and airway responsiveness (30), increased airway inflammation is induced by activation of diverse pro-inflammatory cells including macrophages, neutrophils and eosinophils (31). Treatment with the extract of *P. oleracea *significantly reduced the levels of NO_2_, NO_3_ and MDA while enhanced SOD, CAT and thiol levels compared to the asthmatic group. On the other hand, this condition was reversed in another study using hydro-ethanolic extract of *P. oleracea *in OVA-sensitized rats (9), which was also observed in the present experiment. However, in our previous study (9), the effects of hydro-ethanolic extract of *P. oleracea* and linolenic acid on total and differential WBC counts in the blood as well as serum oxidant/antioxidant biomarkers levels in a rat model of asthma were shown which reflected the influence of the plant and it constituent linolenic acid on systemic inflammation and oxidative stress. However, in the present study, total and differential WBC count, as well as oxidant/antioxidant biomarkers levels in BALF of sensitized animals were assessed which reflected inflammatory and oxidant changes in the lung of sensitized animals rather than systemic changes as reported in our previous paper. In addition, the effect of the extract on lung pathological changes was also examined. Therefore, in this study, the effect of the extract of *P. oleracea* on lung pathological parameters were shown in asthmatic animals.

Our results also showed increased pathological insults including interstitial inflammation, interstitial fibrosis, bleeding, epithelial damage and emphysema, in the lung tissues of asthmatic animals. Previous studies reported the same pathological damages in asthmatic rats (21, 32), which confirms the induction of asthma in sensitized animals. Treatment of asthmatic animals with various concentrations of *P. oleracea *extract resulted in significant protection against most asthma-related lung pathological damages. A previous study showed that ethanol extract of *P. oleracea *attenuated alveolar and interstitial edema, neutrophil infiltration, and hemorrhage in lung tissues of a mouse model of hypoxia-induced pulmonary edema (33), which supports the results of the present study. 

The effects of the two higher concentrations of the extract on most of the measured parameters were more marked than its lowest concentration. The effect of the highest concentration of *P. oleracea *extract on some of the measured parameters was also more pronounced than the medium concentration. Overall, we observed dose-dependent protective effects of *P. oleracea* against inflammation and oxidative state. 

Therefore, the results of the present study along with the above-mentioned studies suggest a potential therapeutic effect for *P. oleracea *against asthma as it decreases lung inflammation and oxidative stress. However, further studies are needed to evaluate the effect of the plant and its constituents in animal models of asthma and asthmatic patients. 

## Conclusion

Our results indicated the preventive effect of the extract of *P. oleracea* on total and differential WBC counts, levels of NO_2_, NO_3_, MDA, SOD, CAT and thiol in BALF and lung pathological insults in asthmatic rats which introduce this herb as a prophylactic remedy against asthma.
